# Quantitative Sensory Testing Reveals Evidence of Altered Pain Processing in Paget’s Disease of Bone

**DOI:** 10.1007/s00223-025-01456-9

**Published:** 2025-12-18

**Authors:** Kathryn Berg, Dervil M. Dockrell, Lesley Colvin, Jonathan C. Y. Tang, Terry Aspray, Elaine Dennison, Hrushikesh Divyateja, Nazim Ghouri, Esther Hanison, Richard Keen, Eugene McCloskey, Terence W. O’Neill, Faizanur Rahman, Mashood Siddiqi, Stephen Tuck, Jane Turton, Stuart H. Ralston

**Affiliations:** 1 Institute of Genetics and Cancer, University of Edinburgh, Western General Hospital, Edinburgh, UK; 2 Division of Population Health and Genomics, Ninewells Hospital and Medical School, University of Dundee, Dundee, UK; 3 Norwich Medical School, University of East Anglia, Norwich, UK; 4 Departments of Clinical Biochemistry, Laboratory Medicine, Diabetes and Endocrinology, Norfolk and Norwich University Hospital NHS Foundation Trust, Norwich Research Park, Norwich, UK; 5 NIHR Newcastle Biomedical Research Centre, Translational Clinical Research Institute, Newcastle University and Newcastle-Upon-Tyne Hospitals NHS Trust, Newcastle Upon Tyne, UK; 6 MRC Lifecourse Epidemiology Centre, University of Southampton, Southampton, UK; 7 NIHR Southampton Biomedical Research Centre, University of Southampton and University Hospital NHS Foundation Trust, Southampton, UK; 8 Chemical Pathology, Nottingham University Hospital, Nottingham, UK; 9 Department of Diabetes and Endocrinology, Queen Elizabeth University Hospital, Glasgow, UK; 10 School of Medicine, University of Glasgow, Glasgow, UK; 11 Metabolic Bone Disease Centre, Royal National Orthopaedic Hospital, London, UK; 12 Division of Clinical Medicine, School of Medicine and Population Health, University of Sheffield, Sheffield, UK; 13 Mellanby Centre for Musculoskeletal Research, Metabolic Bone Centre, Northern General Hospital, Sheffield, England, UK; 14 NIHR Manchester Biomedical Research Centre, Manchester University Foundation NHS Trust, Manchester, UK; 15 Metabolic Medicine and Chemical Pathology, University Hospitals of Leicester NHS Trust, Leicester, UK; 16 Metabolic Bone Diseases, University Hospitals of Liverpool Group, Liverpool, UK; 17 Research and Innovation Centre, Royal National Orthopaedic Hospital, London, UK; 18 Department of Rheumatology, James Cook University Hospital, Middlesbrough, UK; 19 Department of Rheumatology, University Hospital Llandough, Cardiff, UK

**Keywords:** Paget's disease of bone, Pain, Quantitative sensory testing

## Abstract

Pain is the most common symptom of Paget’s disease of bone (PDB), but its underlying mechanisms are poorly understood. Notably, bone pain does not correlate well with metabolic activity or treatment response. This study aimed to assess whether sensory processing is altered in skin overlying Pagetic bone using quantitative sensory testing (QST). We conducted a cross-sectional study of 156 people with PDB attending secondary care referral centres in the UK. We conducted quantitative sensory testing of the skin overlying affected sites and compared the data with unaffected sites as a control. The modalities used were hot and cold rollers, pinprick, vibration and von-Frey filaments to test both spinothalamic and lemniscal pathways. There was a consistent trend for sensory perception to be increased over affected sites versus control sites in the study population. The differences were significant for vibration detection threshold (*p* = 0.009), pain threshold (*p* = 0.010) and both single and multiple pinprick testing methods (both *p* < 0.001). Subgroup analysis revealed similar trends when analysis was restricted to those with pain thought to be due to bone deformity or increased metabolic activity and those with and without musculoskeletal pain. Sensory processing is altered in skin overlying Pagetic bone, independent of current pain symptoms. We speculate that this may be due to abnormalities of bone shape, bone structure or metabolic abnormalities in the affected bone. The mechanisms are unclear but deserve further study.

**Supplementary Information:**

The online version contains supplementary material available at 10.1007/s00223-025-01456-9.

## Introduction

Paget’s disease of bone (PDB) is characterised by focal and disorganised bone remodelling. Although many individuals with PDB are asymptomatic, bone pain remains the most common reason for seeking medical attention [1, 2]. The mechanisms of pain in Paget’s disease are incompletely understood. Previous studies have shown that metabolic activity of PDB does not correlate well with the presence of bone pain, and that in many such patients there is little or no symptomatic response to bisphosphonate therapy [3]. Nonetheless bisphosphonates can be effective at improving bone pain in PDB as evidenced by randomised trials and systematic reviews [4, 5]. Within the bisphosphonates systematic reviews have shown that the response of pain to treatment is better with zoledronic acid [5]. Reflecting this fact, this bisphosphonate is now considered the treatment of first choice for metabolically active Paget’s disease [6]. There are several possible mechanisms for PDB-related pain, including bone deformity, periosteal stretching, microfractures, increased blood flow and osteoclast-mediated acidosis activating pain-sensitive ion channels [7, 8]. In this study we employed quantitative sensory testing (QST) as a means of gaining better understanding of the mechanisms of pain in PDB. Quantitative sensory testing is a non-invasive method of assessing the function of peripheral and central sensory pathways. It has been widely used in the study of neuropathic and musculoskeletal pain conditions, including osteoarthritis, rheumatoid arthritis, and cancer-induced bone pain [9–12]. To date, QST has not been utilised in the study of PDB. Given the potential contribution of altered pain processing in this condition, we used QST to investigate sensory function in individuals with PDB by comparing responses in skin overlying Paget-affected bone to contralateral unaffected sites.

## Subjects and Methods

### Study Subjects

The study was conducted in a subgroup of participants who took part in of the Pain in Paget’s study (PiP). This was a cross-sectional, observational study of 168 patients with PDB attending 12 secondary referral centres from across the UK [2]. Details of recruitment for the study have been reported previously [2]. The QST was performed on 156 (92.9%) of the 168 participants who took part in the PiP study. The remaining twelve were excluded either because they were physically unable to hold a suitable position for testing, or because it was not possible to reach the skin over the affected bone for testing.

### Imaging and Identification of Sites for Sensory Testing

The sites of Paget’s disease had previously been identified by x-ray and radionuclide bone scans as previously described [2]. We created a ‘map’ based on this information to ensure that the QST was undertaken on skin overlying affected bone, as well as to identify an unaffected contralateral or control site. For participants with Paget’s disease of the spine, where a contralateral unaffected site was not possible, we used a site over an unaffected vertebra as a control. For participants with Paget’s disease of the skull where no contralateral site could be identified we used the maxilla or mandible as the control site. Where more than one affected site was identified by imaging, the site that the participant considered the most painful was chosen. If the affected sites were not painful, we chose the most accessible site where there was a suitable contralateral control site. Full details of the sites used are shown in Supplementary Table 1.

We performed a subgroup analysis in subjects who had Pagetic bone deformity and metabolically active disease. Pagetic bone deformity was ascertained clinically. Metabolically active disease was defined to exist in people with pain localised to an affected site where there was biochemical evidence of active disease, reflected by a serum total alkaline phosphatase value or a serum procollagen type I propeptide value above the reference range.

### Quantitative Sensory Testing

The QST protocol developed for this study was a modified version of the protocols used by Rolke et al*.* [13] and Scott et al*.* [12]. Prior to commencing the QST, each participant was briefed on exactly what the procedure involved. All QST was conducted by KB, DD, and EH who had previously undertaken extensive training in the QST procedure used. Each QST session was completed within approximately twenty minutes, and all nine modalities were assessed in a single sitting. For all modalities the individual tests alternated between unaffected and affected sites starting with an unaffected site, until testing for that modality had been completed. A summary of the sensory channels that are thought to be assessed by the different QST modalities is shown in Table 1.

**Table 1:** Summary of sensory channels assessed by quantitative sensory testing

Stimulus	Peripheral sensory channel	Central pathway	QST
Heat pain	C, Aδ	Spinothalamic	Warm thermoroller
Cold pain	C, Aδ	Spinothalamic	Cool thermoroller
Sensation detection Threshold	Aβ	Lemniscal	Von Frey hairs
Vibration	Aβ	Lemniscal	Tuning fork
Mechanical pain	C, Aδ	Spinothalamic	Calibrated pins

Adapted from Hansson et al. [14] and Martland et al. [15]

We used Rolltemp II warm (40 °C) and cool (20 °C) rollers (Somedic SenseLab AB, Sweden) to assess the perception of thermal sensations. The rollers were moved along the skin overlying the affected and unaffected sites. Participants were asked to rate the pain caused by each roller on a Visual Analogue Scale (VAS), ranging from 0 (no pain at all) to 10 (the worst pain they could imagine).

We used a 64Hz tuning fork (Tuning Fork C64 Rydel Seiffer, Bailey Instruments, UK) to measure sensory thresholds for vibration. Each prong of the tuning fork is affixed with a weight upon which an arbitrary scale from 0 to 8 is printed. As the vibration frequency decreases over time, the corresponding number on the scale increases, giving the user a quantitative measure of the point at which vibration was no longer felt. This number is recorded as the Vibration Detection Threshold (VDT). Higher numbers indicate that the testing site is more sensitive to lower vibration frequencies. Each test was repeated up to five times at each site to provide an average score unless the participant reported the same number three times, and a VAS score was recorded. Participants were asked to keep their eyes closed throughout the VDT testing so that they were unaware of the frequencies being tested and recorded above each site. While participants would be aware which site was affected and which was the control, they would not be able to discern which frequency was being applied to the site, reducing detection bias.

We used Aesthesio® sensory evaluators (Linton Instrumentation UK) to assess the Sensation Detection Threshold (SDT) and Pain Threshold (PT) at each site. These are plastic filaments of an increasing thickness which apply a controlled pressure, when pressed against the skin ranging from 0.008g to 300g of force. The SDT was recorded as the pressure at which the participant could first feel the monofilament as force increased, and the PT was recorded as the pressure at which the participant became uncomfortable. A VAS was taken at the PT. As with the VDT, each test was reviewed up to five times at each site to provide an average score unless the participant reported the same number three times. Participants were also asked to close their eyes for SDT and PT testing so that they were unaware which filament/pressure was being administered to each site, to reduce the risk of bias.

Pain sensation was assessed using neurological examination pins (Medipin). We recorded two scores using this technique. A single pinprick (SP) score was recorded on a VAS following a single press of the Medipin on the skin overlying each site. We evaluated temporal summation of pain (TSP) by recording the VAS pain score recorded following five presses of the Medipin in succession on the skin overlying each site. This is considered a human proxy for wind-up of dorsal horn neurons as assessed in animals [16]. Temporal summation scores (TSS) were calculated by subtracting the VAS score reported following the single pinprick testing from the VAS score reported following the wind-up method.

### Biochemistry

Routine biochemistry was measured by standard techniques at the local hospital laboratories. Specialised biochemical markers of bone turnover and cytokines were measured centrally at the Bioanalytical Facility, University of East Anglia. Measurements of the carboxy-terminal telopeptide crosslinks of type I collagen (CTX) were made using an electrochemiluminesence immunoassay (ECLIA) on a Cobas e601 analyser (Roche Diagnostics, Germany). The inter-assay coefficient of variation (CV) for CTX was ≤ 3% between 0.2 and 1.5 µg/L with a sensitivity of 0.01 µg/L. The reference ranges in men and women combined was 0.16–0.85 μg/L. Measurements of amino terminal propeptide of type I collagen (PINP) were also made by ECLIA on a Cobas e601 analyser. The PINP inter-assay CV was ≤ 3% between 20 and 600 µg/L with the sensitivity of 8 µg/L. The reference range for men and women was 15.0–76.3 μg/L. Bone-specific alkaline phosphatase (BAP) was measured using the MicroVue enzyme immunoassay (Quidel, Athens, OH, USA). Inter-assay CV for BAP was ≤ 2.4% up to the concentration of 140 U/L with the lower limit of sensitivity at 0.7 U/L. The BAP reference range for men and women was 11.6–42.7 U/L. Macrophage Colony Stimulating Factor (M-CSF) and Interleukin 6 (IL-6) were measured using Enzyme-Linked Immunosorbent Assays (ELISA) (Quantikine DMC00B and D6050; Bio-techne R&D Systems, Minneapolis, MN, USA.) according to the manufacturer’s instructions. Inter-assay coefficient of variation (CV) for M-CSF was 3.3–7.4% between the assay lower to upper working limits of 11.7–5000 pg/mL. The manufacturer’s reference range in health donors was 180–474 pg/mL. The inter-assay CV for IL-6 was 4.7–8.6% between the assay upper limit of 300 pg/mL and the lower limit of sensitivity at 0.7 pg/mL. The manufacturer’s reference range in healthy donors ranged from 0.7 pg/mL to 13.9 pg/mL.

### Statistical Analysis

Statistical analysis was performed using SPSS version 29. Given that the nine QST modalities assessed were related and represented two principal sensory pathways, we applied a Bonferroni correction to account for multiple comparisons and set the threshold for statistical significance at *p* < 0.025. A paired t-test was used to compare findings between affected and unaffected sites given the relatively large sample size. Possible correlations between QST data, cytokines and biochemical markers of bone remodelling were evaluated using Spearman’s correlation.

## Results

### Characteristics of the Study Population

The clinical characteristics of the study population are shown in Table 2. The average age was 73.7 years with a slight predominance of males and 8.9% had a family history of PDB. Most had monostotic disease. Bone deformity was present in 31.4%, and 7.7% had experienced a previous fracture through Pagetic bone. Musculoskeletal pain was recorded in 111 (71.2%). This was thought to be due to osteoarthritis distant from an affected bone in 48 (30.8%), osteoarthritis neighbouring an affected bone in 10 (6.4%), increased metabolic activity in 15 (9.6%), bone deformity in 11 (7.1%) and neuropathic pain in 10 (6.4%). A variety of other causes of pain accounted of the remainder. Just over half had received previous bisphosphonate treatment for PDB.

**Table 2:** Clinical characteristics of study population

*Demographics*	
Number of individuals	156
Current age	73.7 ± 9.7
Age at diagnosis of PDB	63.6 ± 11.0
Male	89 (57.1%)
Family history of PDB	14 (9.0%)
Current smoker	11 (7.1%)
Alcohol intake (units/week)	6.9 ± 10.2
Body mass Index	26.4 ± 9.3
*Clinical features*	
Previous bisphosphonate for PDB	86 (55.1%)
Monostotic	104 (66.7%)
Number of PDB-affected bones	1 (1–10)
Bone deformity	49 (31.4%)
Hearing Aid with skull involvement	5 (3.2%)
Limb shortening	17 (10.9%)
Osteosarcoma	1 (0.6%)
Previous fracture through Pagetic bone	12 (7.7%)
Spinal stenosis	7 (4.5%)
Presence and causes of musculoskeletal pain	
Musculoskeletal pain present	111 (71.2%)
Pain secondary to osteoarthritis distant from affected site	48 (30.8%)
Pain secondary to osteoarthritis adjacent to affected site	10 (6.4%)
Increased metabolic activity of PDB	15 (9.6%)
Pagetic bone deformity	11 (7.1%)
Neuropathic pain	10 (6.4%)
Other cause	31 (19.8%)
*Biochemistry*	
Serum creatinine (µmol/L)	81.4 ± 25.1
Serum 25(OH)D (nmol/L)	69.1 ± 28.8
Serum total ALP (U/L)	108.8 ± 68.1
Serum BAP (U/L)	29.4 ± 31.2
Serum CTX (μg/L)	0.33 ± 0.23
Serum PINP (μg/L)	73.9 ± 86.0
Serum IL-6 (pg/mL)	3.3 ± 9.5
Serum M-CSF (pg/mL)	423.3 ± 292.1

Values are numbers and % or mean ± SD, except for number of affected bones which is median and range. Reference ranges for serum cytokines and biochemical markers of bone turnover are provided in the methods section. Note that the proportion of patients with different causes of pain add up to more than 100% as some had more than one cause.

### Sites Assessed by Qualitative Sensory Testing

The most common site was the pelvis (43.6% of QST participants), followed by the lumbar spine (14.3%), tibia (10.9%), femur 10.3%), skull (7.7%), humerus (5.1%), and thoracic spine (4.5%). Other sites included the radius (1.3%) scapula, ribs, hands and feet (0.6%). A full list of testing sites can be found in Supplementary Table 1.

### Results of Quantitative Sensory Testing

Sensory thresholds differed significantly between affected and unaffected bone as shown in Table 3. Pain sensitivity, as measured by pinprick stimulation, was consistently increased over affected sites. Both single pinprick and wind-up pinprick stimuli produced higher visual analogue scale (VAS) scores over affected bone (*p* < 0.001 for both). Pain thresholds, assessed using von Frey filaments, were significantly lower over Pagetic bone (*p* = 0.010), and VAS scores for discomfort at pain threshold were also higher (*p* = 0.011).

**Table 3:** Quantitative sensory testing in Paget’s disease

Modality	Site		
	Affected	Unaffected	*p* value
Temperature			
Cool roller VAS	0.21 ± 0.09	0.13 ± 0.08	0.091
Warm roller VAS	0.19 ± 0.08	0.09 ± 0.05	0.049
Vibration			
Vibration detection threshold	3.40 ± 0.18	3.78 ± 0.18	0.009
Vibration VAS	0.37 ± 0.11	0.23 ± 0.08	0.072
Sensation			
Sensation detection threshold	2.23 ± 0.71	1.52 ± 0.66	0.460
Pain			
Pain threshold	146.1 ± 9.7	164.9 ± 10.0	0.010
Visual analogue Scale	3.73 ± 0.22	3.35 ± 0.23	0.011
Single pinprick VAS	2.76 ± 0.21	1.73 ± 0.18	0.000
Multiple pinprick VAS	4.4 ± 0.22	3.7 ± 0.21	0.000
Temporal summation score	1.95 ± 0.18	1.64 ± 0.14	0.064

The values shown are means ± standard error of the mean from 156 subjects. The *p*-values refer to differences between affected and unaffected bone

Vibration detection thresholds were significantly reduced over affected sites (mean 3.40 ± 0.18 vs. 3.78 ± 0.18; *p* = 0.009), suggesting decreased sensitivity overlying Pagetic bone. Warm and cool temperature stimuli showed a trend toward increased sensitivity over affected sites, but these differences did not reach the adjusted threshold for statistical significance (warm roller VAS: *p* = 0.049; cool roller VAS: *p* = 0.091).

These sensory changes were observed irrespective of whether participants reported pain at the time of assessment, indicating that altered processing occurs even in the absence of clinical symptoms.

### Effect of Metabolic Activity, Bone Deformity, and Pain on Quantitative Sensory Testing

We also evaluated a subgroup of 24 participants with pain attributed to metabolically active PDB disease or bone deformity associated with PDB. In this subgroup, 13 participants had test sites of metabolically active disease, 9 had test sites of deformity and 2 had test sites where there was both deformity and metabolically active disease. The QST findings mirrored those in the full cohort. Vibration detection thresholds were lower over affected sites in this subgroup (*p* = 0.041), and temporal summation scores were higher (*p* = 0.032). Pain VAS scores also differed between affected and unaffected sites (*p* = 0.036), although other differences did not reach statistical significance in this smaller sample (Table 4).

**Table 4:** Quantitative Sensory testing of sites with evidence of increased metabolically activity and bone deformity

Modality	Site		
	Affected	Unaffected	*p* value
Temperature			
Cool roller VAS	0.25 ± 0.25	0.08 ± 0.08	0.328
Warm roller VAS	0.25 ± 0.25	0.08 ± 0.08	0.328
Vibration			
Vibration detection threshold	3.08 ± 0.43	3.96 ± 0.46	0.041*
Vibration VAS	0.0 ± 0.0	0.2 ± 0.15	0.170
Sensation			
Sensation detection threshold	0.74 ± 0.24	0.54 ± 0.25	0.358
Pain			
Pain threshold	160.8 ± 25.2	163.8 ± 24.0	0.852
Visual analogue scale	4.08 ± 0.58	2.92 ± 0.52	0.036*
Single pinprick VAS	3.0 ± 0.53	2.08 ± 0.51	0.147
Multiple pinprick VAS	4.98 ± 0.56	4.25 ± 0.61	0.032*
Temporal summation score	2.16 ± 0.52	1.95 ± 0.41	0.717

Values are mean ± sem. The data shown are from 24 sites. The *p*-values refer to differences between affected and unaffected bone

### Relation Between Sensory Testing, Circulating Cytokines and Biochemical Markers of Bone Turnover

We observed no significant correlations between any of the QST measurements over affected or unaffected sites and circulating concentrations of PINP, CTX, total ALP, BALP, IL-6 or M-CSF in the whole population or in the subgroup who had not previously been treated with bisphosphonates (data not shown).

### Relation Between Sensory Testing and the Presence or Absence of Musculoskeletal Pain

The overall pattern of QST results were very similar in 111 participants who reported the presence of musculoskeletal pain and the 44 who did not (data not shown). The only exception was in the temporal summation scores which were significantly higher over both affected and unaffected bone in people who reported musculoskeletal pain as compared with those who did not (Table 5). As in the whole study population the scores were significantly higher over affected compared with unaffected bone in both subgroups.

**Table 5:** Temporal summation score in relation to presence or absence of pain

Participants with pain (*n* = 112)			Participants without pain (*n* = 44)		
Affected	Unaffected		Affected	Unaffected	
2.30 ± 0.22	1.94 ± 0.18	*p* < 0.001	1.07 ± 0.28	0.88 ± 0.18	*p* = 0.001

Values are means ± sem. The *p*-values refer to differences between affected and unaffected bone within the groups of participants with and without pain. The absolute scores were significantly higher in those with pain (*p* < 0.001 pain vs. no pain)

## Discussion

To our knowledge, this is the first study to assess sensory processing in Paget’s disease of bone (PDB) using quantitative sensory testing. Our findings show that the skin overlying Paget-affected bone exhibits increased sensitivity to mechanical stimuli and reduced pain thresholds compared to unaffected contralateral sites. These differences were observed regardless of the presence of musculoskeletal pain, suggesting that altered sensory processing may occur even in the absence of clinical symptoms. The pain thresholds were significantly lower and pain scores significantly higher over the affected site, suggesting that sensory processing may be altered, possibly influenced by abnormalities in bone shape, bone structure, blood flow or by metabolic activity in underlying bone.

The fact that these features were observed when testing the skin over affected sites raises the possibility of peripheral sensitisation, but a feature against that is the fact that we did not observe differences in perception of heat and cold between sites. Furthermore, it is known that central sensitisation can also be manifest by changes in sensory perception over affected sites [17]. A degree of central sensitisation would be expected to occur given that 71% of the cohort experienced musculoskeletal pain, even though this was most commonly due to causes other than Paget’s disease, the most common of which was osteoarthritis.

With the exception of the Temporal Summation Scores, there were no significant differences found in QST between the subgroup reporting musculoskeletal pain, and the subgroup who reported that they were pain-free. Previous literature has suggested that where a higher TSS is observed, a greater degree of central sensitisation is present, which may explain why differences were found between the pain-reporting and pain-free groups. Identifying higher levels of central sensitisation in patients experiencing musculoskeletal pain could influence future treatment strategies and outcomes.

Quantitative sensory testing is widely used in the assessment and diagnosis of neuropathic pain, but over recent years it has been used to assess pain mechanisms conditions such as rheumatoid arthritis and osteoarthritis [18–20]. For example, Kosek et al. observed reduced pain thresholds both at affected joints and at remote sites in people with osteoarthritis, which normalised after joint replacement, indicating the presence of peripheral and central sensitisation. In the study of Lee and colleagues, low pain thresholds were associated with several measures of inflammatory activity including Clinical Disease Activity Index, tender joint counts and patient global assessment scores [20]. The study of Scott and colleagues investigated QST in a series of 23 individuals with bone metastases and reported significantly increased sensitivity to mechanical stimulation between affected and control sites but noticed no difference regarding thermal stimuli [21]. In this study that pain thresholds as assessed by QST increased above the site of bone pain following treatment with radiotherapy. This is of interest since cancer-induced bone pain (CIBP) is described similarly to PDB-related bone pain, in that it is dull, persistent, and worsens over time [22]. Future research into the use of QST in PDB might consider utilising a pre- and post- bisphosphonate treatment approach to assess the role of bone turnover in pain threshold levels.

While the exact mechanisms driving this altered sensory processing remain unclear, several hypotheses can be considered. Enlargement and deformation of Pagetic bone may lead to mechanical stimulation or stretch of pain-sensitive structures such as the periosteum. Osteoclast-mediated bone resorption has also been shown to lower local pH and activate acid-sensing ion channels, which can stimulate nociceptors. Increased vascularity of affected bone could also play a role.

The strengths of this study include the large and well-characterised sample, the systematic application of a QST protocol, and the careful localisation of affected and unaffected sites using imaging. However, several limitations must be acknowledged. Participants were not blinded to the location of their affected bone, which may have introduced response bias in subjective sensory assessments such as pinprick and temperature VAS ratings. Nevertheless, we observed consistent differences in vibration detection, and sensation detection, which are less susceptible to bias as these were conducted with participants’ eyes closed. Secondly, factors such as medication use, mood, or comorbid conditions may have influenced sensory perception and were not controlled for in this analysis.

In conclusion, we have clearly demonstrated altered sensation in the skin overlying affected bone in PDB which doesn’t appear to be influenced solely by increased metabolic activity or deformity. These findings indicate that pharmacological therapies targeted at neural transmission pathways may be helpful for the management of pain in patients with PDB if the response to bisphosphonate therapy is inadequate.

## Supplementary Information

Below is the link to the electronic supplementary material.

Supplementary Material 1

## Declarations

### Human and Animal Welfare

Ethical approval for the PiP study was obtained from the West of Scotland Research Ethics Committee 3 (18/WS/0236). The study was conducted in keeping with the principles of Good Clinical Practice (GCP) and all participants provided written and informed consent.
