# Supplementary material for: Quantitative Sensory Testing Reveals Evidence of Altered Pain Processing in Paget’s Disease of Bone
Source: Calcif Tissue Int. 2025 Dec 18;117(1):2. doi: 10.1007/s00223-025-01456-9 (PMC12714786; doi:10.1007/s00223-025-01456-9)
Supplement: Supplementary file 1 — Supplementary Material 1 [file 223_2025_1456_MOESM1_ESM.docx]

**Supplementary Material**

**Quantitative sensory testing reveals evidence of altered pain processing in Paget’s Disease of Bone**

Kathryn Berg^1^, Dervil Dockrell^1^, Lesley Colvin ^2^, Jonathan C Y Tang ^3,4^, Terry Aspray^5^ Elaine Dennison^6^, Hrushikesh Divyateja^7^, Nazim Ghouri ^8,9^, Esther Hanison^10^, Richard Keen^10^, Eugene McCloskey^11^, Terence W O'Neill^12^, Faizanur Rahman^13^, Mashood Siddiqi^14^, Stephen Tuck^15^, Jane Turton^16^, Stuart H Ralston^1.^

^1^ Institute of Genetics and Cancer, University of Edinburgh, Western General Hospital, Edinburgh, UK

^2^ Division of Population Health and Genomics, Ninewells Hospital and Medical School, University of Dundee, Dundee, UK

^3^ Norwich Medical School, University of East Anglia, Norwich, UK

^4^ Departments of Clinical Biochemistry, Laboratory Medicine, Diabetes and Endocrinology, Norfolk and Norwich University Hospital NHS Foundation Trust, Norwich Research Park, Norwich, UK.

^5^ NIHR Newcastle Biomedical Research Centre, Translational Clinical Research Institute, Newcastle University and Newcastle-upon-Tyne Hospitals NHS Trust, Newcastle upon Tyne, UK

^6^ MRC Lifecourse Epidemiology Centre, University of Southampton, Southampton, UK

^7^ Nottingham University Hospital, Chemical Pathology, Nottingham, UK

^8^ Queen Elizabeth University Hospital, Department of Diabetes and Endocrinology, Glasgow, UK

^9^ School of Medicine, University of Glasgow, Glasgow, UK

^10^ Royal National Orthopaedic Hospital, Metabolic Bone Disease Centre, London, UK

^11^ Clinical Medicine, School of Medicine and Population Health, Metabolic Bone Centre, Northern General Hospital, UK

^12^ University of Manchester, Manchester Institute for Collaborative Research on Ageing, Manchester, UK

^13^ University Hospitals of Leicester NHS Trust, Metabolic Medicine & Chemical Pathology, Leicester, UK.

^14^ Liverpool University Hospitals Foundation Trust, Aintree University Hospital, Liverpool, UK

^15^ James Cook University Hospital, Department of Rheumatology, Middlesbrough, UK

^16^ University Hospital Llandough, Department of Rheumatology, Cardiff, UK

**Supplementary Table 1. Sites used for quantitative sensory testing**

| PiP Study ID Number | Affected Site | Control Site |
| --- | --- | --- |
| PIP001001 | Right Hip | Left Hip |
| PIP001002 | Right Hip | Left Hip |
| PIP001003 | Lumbar Spine | Thoracic Spine |
| PIP001004 | Right Tibia | Left Tibia |
| PIP001005 | Left Hip | Right Hip |
| PIP001006 | Right Hip | Left Hip |
| PIP001007 | Left Hip | Right Hip |
| PIP001008 | Left Hip | Right Hip |
| PIP001009 | Right Femur | Left Femur |
| PIP001010 | Left Hip | Right Hip |
| PIP001011 | Right Tibia | Left Tibia |
| PIP001012 | Right Hip | Left Hip |
| PIP001013 | Right Hip | Left Hip |
| PIP001014 | Right Hip | Left Hip |
| PIP001015 | Left Hip | Right Hip |
| PIP001016 | Right Humerus | Left Humerus |
| PIP001017 | Left Hip | Right Hip |
| PIP001019 | Skull | Skull |
| PIP001020 | Lumbar Spine | Thoracic Spine |
| PIP001021 | Left Hip | Right Hip |
| PIP001022 | Lumbar Spine | Thoracic Spine |
| PIP001023 | Right Hip | Left Hip |
| PIP001024 | Skull | Skull |
| PIP001025 | Left Hip | Right Hip |
| PIP001026 | Right Tibia | Left Tibia |
| PIP001027 | Right Hip | Left Hip |
| PIP001028 | Right Scapula | Left Scapula |
| PIP001029 | Right Hip | Left Hip |
| PIP001030 | Right Ribs | Left Ribs |
| PIP001031 | Left Hip | Right Hip |
| PIP001032 | Left Hip | Right Hip |
| PIP001034 | Left Hip | Right Hip |
| PIP001035 | Left Tibia | Right Tibia |
| PIP001037 | Lumbar Spine | Thoracic Spine |
| PIP001038 | Left Hand | Right Hand |
| PIP001039 | Right Hip | Left Hip |
| PIP001040 | Right Hip | Left Hip |
| PIP001041 | Right Hip | Left Hip |
| PIP001042 | Right Femur | Left Femur |
| PIP001043 | Right Hip | Left Hip |
| PIP002001 | Left Hip | Right Hip |
| PIP002002 | Right Hip | Left Hip |
| PIP002003 | Right Hip | Left Hip |
| PIP002004 | Lumbar Spine | Thoracic Spine |
| PIP002005 | Left Tibia | Left Femur |
| PIP002006 | Left Tibia | Right Tibia |
| PIP002008 | Left Hip | Right Hip |
| PIP002009 | Right Tibia | Left Tibia |
| PIP002010 | Left Humerus | Right Humerus |
| PIP002011 | Left Hip | Right Hip |
| PIP003001 | Right Tibia | Left Tibia |
| PIP003002 | Right Hip | Left Hip |
| PIP003004 | Left Hip | Right Hip |
| PIP003005 | Thoracic Spine | Cervical Spine |
| PIP003006 | Left Femur | Right Femur |
| PIP003007 | Right Femur | Left Femur |
| PIP003008 | Lumbar Spine | Thoracic Spine |
| PIP003009 | Right Femur | Left Femur |
| PIP003010 | Lumbar Spine | Thoracic Spine |
| PIP003011 | Right Femur | Left Femur |
| PIP003012 | Lumbar Spine | Thoracic Spine |
| PIP003017 | Skull | Skull |
| PIP003018 | Lumbar Spine | Thoracic Spine |
| PIP003019 | Right Femur | Left Femur |
| PIP003020 | Lumbar Spine | Thoracic Spine |
| PIP005001 | Right Hip | Left Hip |
| PIP005002 | Thoracic Spine | Lumbar Spine |
| PIP005003 | Thoracic Spine | Lumbar Spine |
| PIP005004 | Left Femur | Right Femur |
| PIP005005 | Left Hip | Right Hip |
| PIP005007 | Thoracic Spine | Cervical Spine |
| PIP005008 | Lumbar Spine | Thoracic Spine |
| PIP005009 | Lumbar Spine | Thoracic Spine |
| PIP006001 | Right Hip | Left Hip |
| PIP006002 | Other | Lumbar Spine |
| PIP006003 | Right Foot | Left Foot |
| PIP006004 | Right Humerus | Left Humerus |
| PIP006005 | Left Femur | Right Femur |
| PIP006006 | Lumbar Spine | Thoracic Spine |
| PIP008001 | Thoracic Spine | Thoracic Spine |
| PIP008002 | Right Femur | Left Femur |
| PIP008003 | Lumbar Spine | Thoracic Spine |
| PIP008004 | Left Hip | Right Hip |
| pip008005 | Lumbar Spine | Thoracic Spine |
| PIP008006 | Right Tibia | Left Tibia |
| PIP008007 | Right Hip | Left Hip |
| PIP008008 | Right Tibia | Left Tibia |
| PIP008009 | Left Hip | Right Hip |
| PIP008010 | Right Tibia | Left Tibia |
| PIP008012 | Left Femur | Right Femur |
| PIP008013 | Right Hip | Left Hip |
| PIP008014 | Left Femur | Right Femur |
| PiP008015 | Right Hip | Left Hip |
| PIP008016 | Lumbar Spine | Thoracic Spine |
| PIP008017 | Left Hip | Right Hip |
| PIP008018 | Lumbar Spine | Thoracic Spine |
| PIP008019 | Left Hip | Right Hip |
| PIP009003 | Right Hip | Left Hip |
| PIP010001 | Left Hip | Right Hip |
| PIP010002 | Right Tibia | Left Tibia |
| PIP010003 | Skull | Skull |
| PIP010004 | Skull | Skull |
| PIP010005 (TM) | Left Hip | Right Scapula |
| PIP010006 | Left Tibia | Right Tibia |
| PIP010007 | Skull | Skull |
| PIP010008 (PD) | Right Hip | Left Hip |
| PIP010009 | Right Hip | Left Hip |
| PIP010010 | Right Tibia | Left Tibia |
| PIP010011 | Right Femur | Left Femur |
| PIP010012 | Skull | Skull |
| PIP010014 | Right Hip | Left Hip |
| PIP010015 | Left Hip | Right Hip |
| PIP010016 | Skull | Skull |
| PIP010017 | Right Hip | Left Hip |
| PIP010018 | Right Femur | Left Femur |
| PIP010019 | Right Hip | Left Hip |
| PIP010020 | Left Hip | Right Hip |
| PIP010021 | Right Hip | Left Hip |
| PIP010022 | Right Tibia | Left Tibia |
| PIP011001 | Skull | Left Mandible |
| PIP011002 | Lumbar Spine | Thoracic Spine |
| PIP011003 | Skull | Skull |
| PIP011004 | Right Humerus | Left Humerus |
| PIP011005 | Left Humerus | Right Humerus |
| PIP011006 | Skull | Left Mandible |
| PIP011007 | Other | Thoracic Spine |
| PIP011008 | Left Hip | Right Hip |
| PIP011009 | Lumbar Spine | Thoracic Spine |
| PIP011010 | Right Femur | Left Femur |
| PIP011011 | Thoracic Spine | Thoracic Spine |
| PIP011012 | Skull | Left Mandible |
| PIP011013 | Right Humerus | Left Humerus |
| PIP011014 | Right Hip | Left Hip |
| PIP011015 | Left Hip | Right Hip |
| PIP012001 | Sacrum | Lumbar Spine |
| PIP012002 | Right Tibia | Left Tibia |
| PIP012003 | Sacrum | Lumbar Spine |
| PIP012004 | Right Hip | Left Hip |
| PIP012005 | Left Radius | Right Radius |
| PIP012006 | Left Hip | Right Hip |
| PIP012007 | Right TIbia | Left Tibia |
| PIP012008 | Right Hip | Left Hip |
| PIP012009 | Right Hip | Left Hip |
| PIP013001 | Right Hip | Left Hip |
| PIP013002 | Right Hip | Left Hip |
| PIP013004 | Thoracic Spine | Thoracic Spine |
| PIP013005 | Right Femur | Left Femur |
| PIP013006 | Left Radius | Right Radius |
| PIP013007 | Right Hip | Left Hip |
| PIP013008 | Right Humerus | Right Humerus |
| PIP014001 | Left Hip | Right Hip |
| PIP014002 | Lumbar Spine | Thoracic Spine |
| PIP014003 | Right Hip | Left Hip |
| PIP014005 | Lumbar Spine | Cervical Spine |
| PIP014006 | Right Humerus | Left Humerus |
| PIP014007 | Lumbar Spine | Thoracic Spine |
